# Biological and molecular characterization of fEg-Eco19, a lytic bacteriophage active against an antibiotic-resistant clinical *Escherichia coli* isolate

**DOI:** 10.1007/s00705-022-05426-6

**Published:** 2022-04-10

**Authors:** Shimaa Badawy, Zakaria A. M. Baka, Mohamed I. Abou-Dobara, Ahmed K. A. El-Sayed, Mikael Skurnik

**Affiliations:** 1 Department of Bacteriology and Immunology, Medicum, Human Microbiome Research Program, Faculty of Medicine, University of Helsinki, 00014 UH Helsinki, Finland; 2 Department of Botany and Microbiology, Faculty of Science, Damietta University, New Damietta, 34517 Egypt; 3 Division of Clinical Microbiology, Helsinki University Hospital, HUSLAB, 00290 Helsinki, Finland

## Abstract

Characterization of bacteriophages facilitates better understanding of their biology, host specificity, genomic diversity, and adaptation to their bacterial hosts. This, in turn, is important for the exploitation of phages for therapeutic purposes, as the use of uncharacterized phages may lead to treatment failure. The present study describes the isolation and characterization of a bacteriophage effective against the important clinical pathogen *Escherichia coli*, which shows increasing accumulation of antibiotic resistance. Phage fEg-Eco19, which is specific for a clinical *E. coli* strain, was isolated from an Egyptian sewage sample. Phage fEg-Eco19 formed clear, sharp-edged, round plaques. Electron microscopy showed that the isolated phage is tailed and therefore belongs to the order *Caudovirales*, and morphologically, it resembles siphoviruses. The diameter of the icosahedral head of fEg-Eco19 is 68 ± 2 nm, and the non-contractile tail length and diameter are 118 ± 0.2 and 13 ± 0.6 nm, respectively. The host range of the phage was found to be narrow, as it infected only two out of 137 clinical *E. coli* strains tested. The phage genome is 45,805 bp in length with a GC content of 50.3% and contains 76 predicted genes. Comparison of predicted and experimental restriction digestion patterns allowed rough mapping of the physical ends of the phage genome, which was confirmed using the PhageTerm tool. Annotation of the predicted genes revealed gene products belonging to several functional groups, including regulatory proteins, DNA packaging and phage structural proteins, host lysis proteins, and proteins involved in DNA/RNA metabolism and replication.

**Supplementary Information:**

The online version contains supplementary material available at 10.1007/s00705-022-05426-6.

## Introduction

*Escherichia coli* is a Gram-negative, non-endospore-forming bacterium that is motile by means of peritrichous flagella [1]. It belongs to the family *Enterobacteriaceae* and as a pathogen causes serious urinary and gastrointestinal infections in humans [2, 3]. Furthermore, *E. coli* is used as a contamination indicator for water, food, and agricultural products [4]. It can cause hospital- and community-acquired infections, resulting in diarrhea, meningitis, urinary tract infections (UTIs), bacteremia, pneumonia, surgical-site infections, and sepsis [3]. It can also cause food-borne infections, such as those caused by enterohemorrhagic *E. coli* (EHEC) or Shiga-toxin-producing *E. coli* (STEC). STEC can be acquired by ingestion of contaminated food or water, or through contact with animals or their environment [5]. The worldwide increase in the rate of resistance to carbapenem and colistin among members of the family *Enterobacteriaceae* [6], especially in developing countries [7], is a serious threat to their therapeutic use. In addition, antibiotic resistance in foodborne *E. coli* remains a public health concern and is responsible for severe infections in humans [8]. The changing patterns of antimicrobial resistance and shortage of novel classes of antibiotics [9] have contributed to a demand for new antibacterial agents. This has occurred despite the introduction of carbapenem (imipenem and meropenem) and colistin (polymyxin B), which are the last-resort antibiotics for treatment of Gram-negative bacterial infections. Resistant strains against both antibiotics have been widely reported in clinical settings [10, 11]. Moreover, pan-resistant strains, resistant to all available antibiotics, have been encountered [12]. Due to this threat, phage therapy is being developed and used as an alternative treatment to manage infections caused by multidrug-resistant (MDR) pathogens [13–15]. Compared to antibiotics, phages have several advantages that enable them to be good therapeutic agents. Phages are generally considered safe, as they do not infect animal or plant cells, they are highly specific for a particular host, they replicate only at the site of infection where their target bacteria are present, and they very effectively lyse only the target pathogen and not the bacteria of normal microbiome. The mechanism by which they kill bacteria is different from that of antibiotics, so they are also effective against MDR bacteria. Furthermore, phage propagation is inexpensive, phages are ubiquitous in nature, and side effects are uncommon in phage therapy [16–18].

Phage therapy, the use of lytic bacteriophages to combat bacterial infectious diseases, is regarded as a promising alternative to the use of antimicrobial agents [19, 20]. Bacteriophages and their derived enzymes can be seen as an appealing alternative for treatment of drug-resistant infections [21]. Phage therapy is a realistic complement to antibiotics, especially now that recent modifications to ubiquitous phages have made them more controllable. There is still a need for a better understanding of phage therapy, however, before it can be adopted widely [22, 23]. In this work, we characterized an *Escherichia* phage isolated from an Egyptian sewage sample.

## Materials and methods

### Bacterial strains

The bacterial strains used for phage isolation and host range determination are listed in Supplementary Table S1. The bacteria were cultured in lysogeny broth (LB) or on LB agar (LA) plates as described previously [24].

### Phage isolation and purification

*E. coli*-specific bacteriophages were enriched as described previously [25], with some alterations. Sewage water samples used as sources of phages were collected during winter 2018 from four El-Rezka and El-Korama drainages, Dakahlia Governorate, Egypt. Five drainage samples of each location were collected in sterile 50-mL polypropylene tubes. The samples were centrifuged at 5,000 rpm for 15 min at 4°C, and the supernatants were filtered through 0.45-µm filters (Minisart® Sartorius, Göttingen, Germany) to remove any remaining bacteria, and then combined together. Thirteen clinical *E. coli* strains (Supplementary Table S1, indicated in bold) were grown separately at 37°C for 16 h. Two parallel pools, representing six and seven strains, respectively, were prepared by combining 100-µL aliquots of the cultures. To these pools, one ml of the filtered Egyptian sewage mixture was added, followed by 9 ml of LB. These cultures were incubated for 16 h on a rocking platform at 37°C to enrich any *E. coli*-specific phages present in the sewage samples. To kill the bacteria, chloroform (0.2 ml for every 3 ml of culture) was added to the tubes, followed by mixing on the rocking platform for 20 min at 22°C. The lysates were then centrifuged at 5,000 rpm for 10 min at 4°C, and the supernatants were passed through 0.45-µm filters (Minisart® Sartorius). The resulting enriched lysates were stored at 4°C and used to isolate *E. coli* specific-phages. Phage isolation, titration, plaque purification, and preparation of phage stocks were carried out as described previously [24].

### Transmission electron microscopy

Phage particles were concentrated by centrifugation for 90 min at full speed (16,000 × *g*) in an Eppendorf centrifuge (5415R, rotor model 3328, Enfield, NJ, USA), and the pelleted phages were resuspended in 200 µL of 0.1 M ammonium acetate, pH 7.0. Three 3-µL aliquots of the high-titer phage preparations (10^10^–10^13^ plaque-forming units/mL) were pipetted on carbon-coated copper grids. After allowing the phages to adsorb for one minute, the grids were stained with 2% uranyl acetate (pH 4.2) for 30 s. The grids were then examined for phages using a transmission electron microscope (JEOL JEM-1400, Tokyo, Japan) at 80 kV beam voltage, equipped with a Gatan Orius SC 1000B camera (Gatan Inc., Pleasanton, CA, USA). The heads and tails of 5 to 10 individual phage particles were measured and used to calculate the averages and standard errors for the dimensions.

### Phage host range determination

The host range of fEg-Eco19 was tested on 152 strains, including 137 *E. coli* strains, 10 *Staphylococcus aureus* strains, two *Pseudomonas aeruginosa* strains, two *Acinetobacter baumannii* strains*,* and one *Klebsiella pneumoniae* strain. The phage sensitivity was assayed using the Bioscreen C system (Growth Curves, Helsinki, Finland), in which bacterial growth is measured continuously by vertical photometry (optical density) at 600 nm using a computerized incubator. This method has several advantages: it is rapid, requires little technician time, allows frequent measurement of the growth of the bacteria, processes the data, and provides a printout of the results as growth curves. One-hundred-µL aliquots of 1:500-diluted overnight bacterial cultures were distributed to the wells of honeycomb plates. The plates were then incubated at 37°C with continuous shaking, and growth was monitored for 16 h with 1-h measurement intervals. Each reading was preceded by a 5-s break in the shaking cycle, and the turbidity was recorded using a 600-nm filter. Each strain was tested in three replicates, and the mean OD_600_ was calculated. Bacterial growth curves were plotted as OD_600_ readings versus time [26].

### Phage and bacterial DNA extraction

Phage DNA was extracted using the phenol chloroform method [27], with some previously described modifications [24]. The bacterial genomic DNA was isolated and purified using a JetFlex Genomic DNA Purification Kit (Thermo Fisher Scientific, Waltham, MA, USA), following the manufacturer’s instructions. The quality and quantity of DNA was determined using a NanoDrop spectrophotometer (ND-1000, Wilmington, DE, USA), a Qubit 2.0 fluorometer (Invitrogen, CA, USA) with a Qubit dsDNA BR Assay Kit (Thermo Fisher Scientific), and agarose gel electrophoresis [28].

### Phage genome sequencing, assembly, and bioinformatics

Phage genome sequencing, assembly, and bioinformatics were performed as described previously [24]. A phylogenetic tree based on the phage proteome was generated using VIPTree [29]. The termini of the phage genome were identified using the Phage Term program [30].

### Restriction enzyme digestion

The purified phage DNA was digested with the restriction endonucleases EcoRI, NsiI, SmaI, SalI, NruI (Thermo Fischer Scientific), ClaI, AflII, and BbvCI (New England Biolabs), which were predicted to produce the best-resolved restriction fragment patterns. The restriction enzyme digestions, using ca. 300 ng of phage DNA, were carried out according to manufacturers’ instructions in a final volume of 10 µL. The restriction fragments were separated by electrophoresis in a 1% (w/v) agarose gel containing 0.005% (w/v) of Midori green. The restriction fragment bands were visualized using a Bio-Rad GelDoc XR+ imaging system.

### Accession number

The annotated nucleotide sequence of the phage fEg-Eco19 genome was deposited in the GenBank database under the accession number OL539727.

## Results and discussion

### Isolation of phages

Enrichment of *E. coli*-specific bacteriophages was carried out with two parallel pools, containing six and seven *E. coli* strains, respectively (Supplementary Table S1), and the sterile-filtered enriched lysates were tested for phages using each *E. coli* strain individually as an indicator bacterium on soft-agar plates. Phage activity was detected only against *E. coli* strain #5521.

### Plaque morphology of the isolated phage

**Fig. 1:**
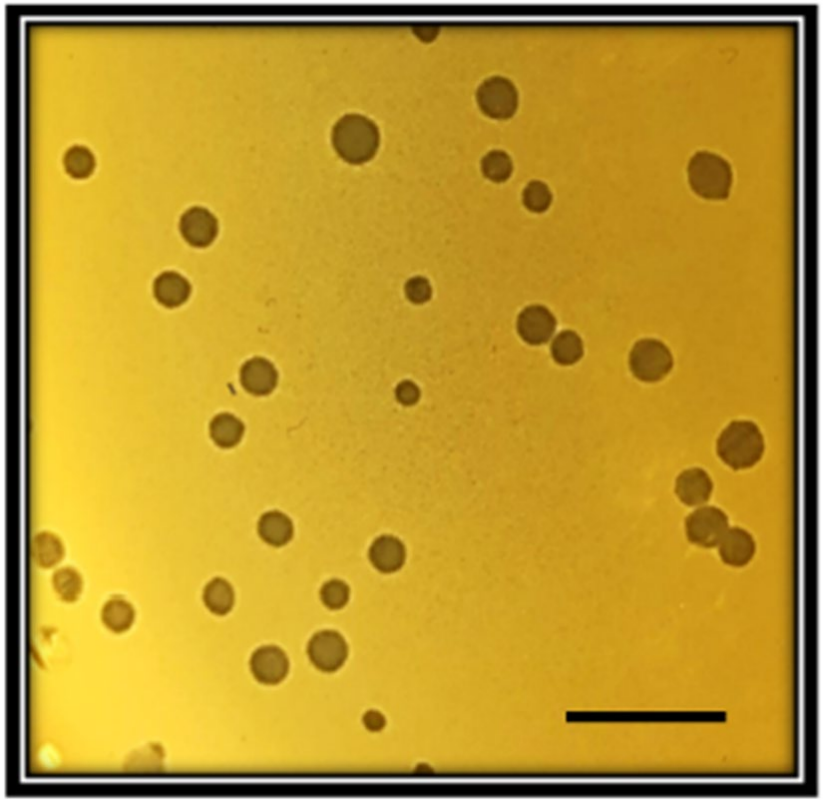
Plaque morphology of phage fEgEco-19 grown for 16 h on an *E. coli* #5521 lawn on an LA plate at 37°C. The scale bar is 1 cm.

Single-plaque purification was performed at least three times to obtain pure phage stocks to be used for phage characterization. The phage that was active against strain #5521, named "fEg-Eco19", formed round, clear, sharp plaques (Fig. 1).

### Phage morphology

**Fig. 2:**
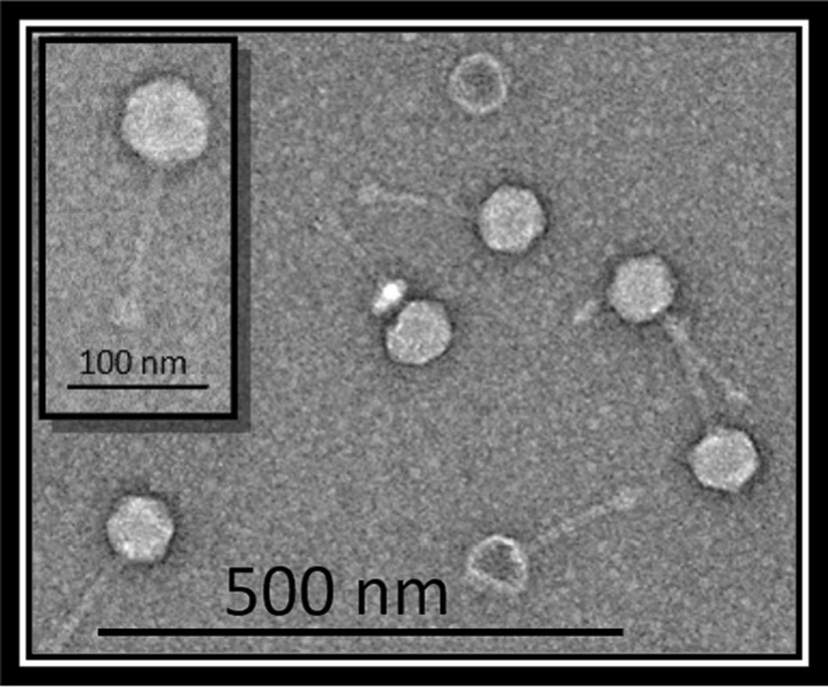
Transmission electron micrograph of fEg-Eco19 negatively stained with 2% uranyl acetate

Transmission electron microscopy revealed that *E. coli* phage fEgEco-19 (Fig. 2) has a siphovirus morphology, as it possess an icosahedral head of 68 ± 2 nm in diameter as well as a long non-contractile tail of 118 ± 0.2 nm in length and 13 ± 0.6 nm in width (Table 1).

**Table 1:** Overview of the morphological features of phage fEG-Eco19. The phage dimensions were measured by TEM and represent the mean value obtained from at least 10 particles.

Bacteriophage	Morphology	Head		Tail		
		Shape	Capsid size (nm)	Type	length (nm)	Width (nm)
fEg-Eco19	Siphovirus	Icosahedral	68 ± 2	Long non- contractile	118 ± 0.2	13 ± 0.6

### Phage host range

**Fig. 3:**
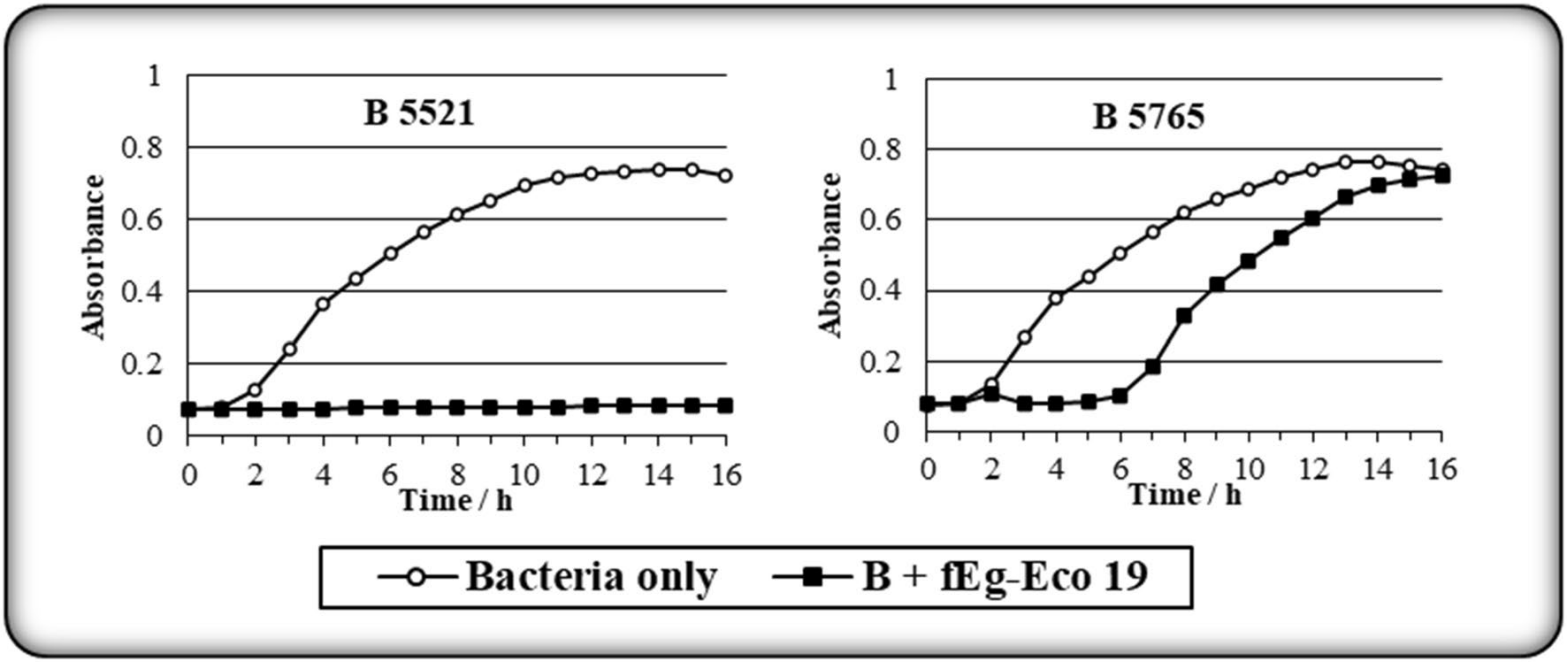
Bacterial growth patterns for host range determination using the Bioscreen C system, in which growth is measured continuously by vertical photometry (optical density) at 600 nm. The results revealed that this phage is highly specific, as it infected only *E. coli* strains #5521 and #5765.

The host range of phage fEg-Eco19 was investigated using 137 *E. coli* strains and 15 strains representing other species (Supplementary Table S1), using the Bioscreen C method, in which the growth is measured continuously by vertical photometry (optical density) at 600 nm. Bacterial growth curves (Fig. 3) revealed that the phage infected only *E. coli* strains #5521 and #5765. Both of these are clinical strains. While strain #5521 is a blood culture isolate that is sensitive to most antibiotics, #5765 is an ESBL strain isolated from a urine sample. The growth curves of the phage-infected cultures showed that spontaneous phage-resistant mutants of #5765 started to grow after the 6 h time point. No such growth was observed for #5521, suggesting that these strains do not share a common phage receptor, and the plaques on #5765 were similar to those on #5521. Further studies are needed to identify the receptors.

### Characterization of the phage genome

**Fig. 4:**
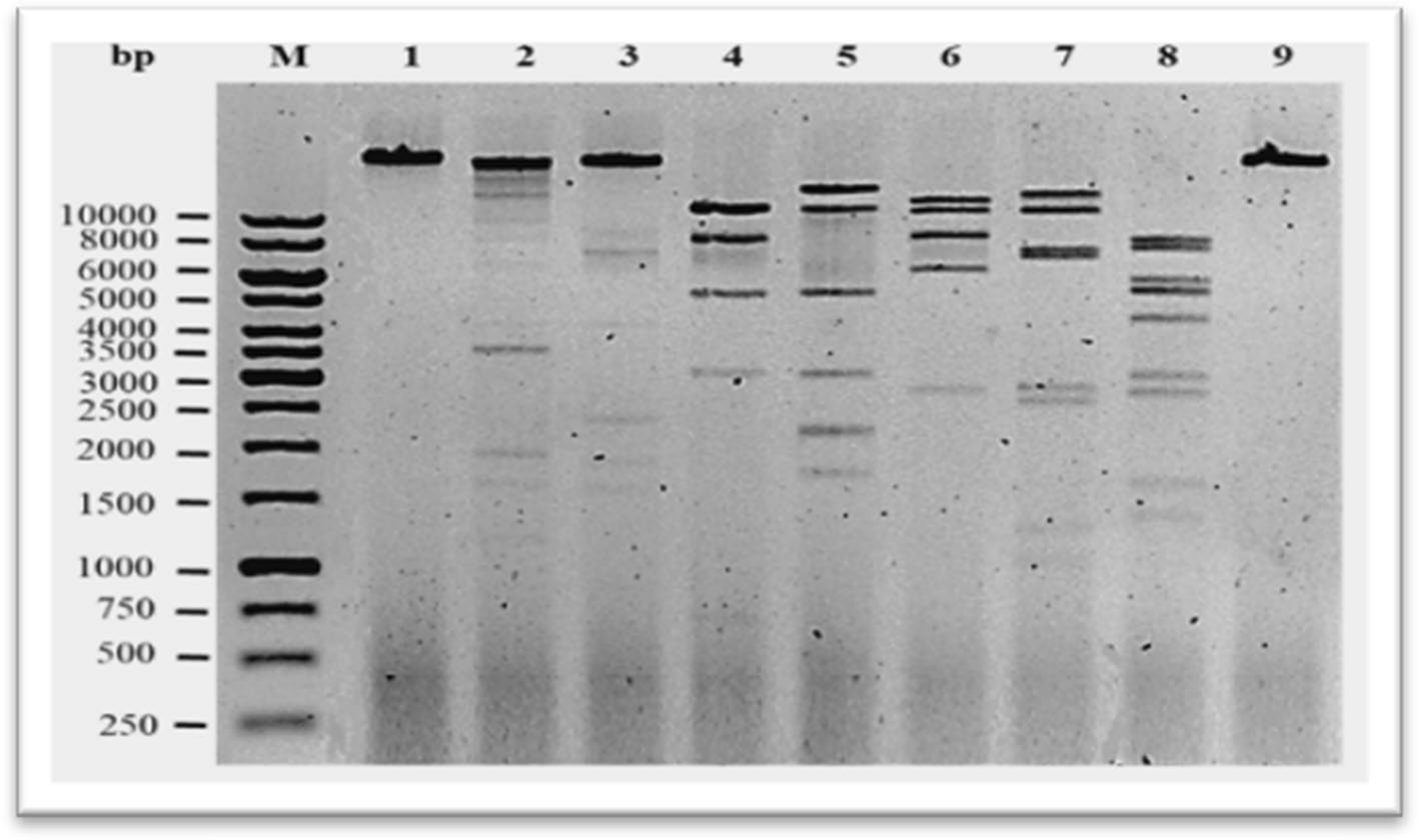
Agarose gel electrophoresis analysis of restriction-enzyme-digested fEg-Eco1*9* DNA. Phage genomic DNA was digested with EcoRI (lane 2), Nsil (lane 3), SmaI (lane 4), SalI (lane 5), NruI (lane 6), ClaI (lane 7), AflII (lane 8), and BbvCI (lane 9). Lane 1, undigested DNA. Lane M, 1-kb DNA ladder

Assembly of the Illumina sequence reads of phage fEgEco-19 revealed that it has a genome of about 45 kb. Of the total sequence reads, 98.8 % mapped to the 45-kb contig, indicating that it represents the complete phage genome. The fEg-Eco19 genome is 45,805 bp in size with a GC-content of 50.2%. PhageTerm analysis allowed the identification of the physical genome termini and revealed that fEgEco-19 uses the phage P1-type headful packaging mechanism, starting from a *pac* site (Supplementary Fig. S1). Phages that use a headful packaging mechanism typically generate a concatemer containing several copies of their genome. The phage terminase initiates packaging of the genome concatemer at the specific *pac* site, and packaging is terminated at variable positions when the phage head becomes full. This leads to capsids containing circularly permuted genomes with somewhat random termini used to circularize the phage genome through recombination after injection into the host cell [30]. In PhageTerm analysis, a slight increase in read start coverage is expected in the region after the *pac* site peak where the second cut is made, since starts in this region will be present in many phage particles. Since packaging is directional and no precise cut is made upon termination of packaging, a peak is expected only in a single orientation, which also informs us about the direction of packaging [30]. Restriction enzyme digestions were used to confirm the PhageTerm-predicted physical ends of the phage (Fig. 4).

The experimental restriction enzyme digestion patterns of phage fEG-Eco19 DNA (Fig. 4) were in perfect agreement with *in silico*-predicted restriction digestion fragment sizes, confirming that the genome sequence was correctly organized based on the identification of the physical ends by PhageTerm analysis:

**EcoRI** (40224, 3565, 1155 left end, 762 right end, 99)

**NsiI** (34390, 5641 right end, 2354, 1848 left end, 1572)

**SmaI** (11409, 11224, 8310, 5862 right end, 5239, 3099 left end, 698)

**SalI** (14431, 11244 left end, 5290, 5077 right end, 3077, 2236, 2192, 1745, 513)

**NruI** (11937, 10679, 8264, 6109, 5971 right end, 2748 left end, 97)

**ClaI** (13004, 10790, 7310, 6919, 2859, 2589, 1032, 908 right end, 358 left end)

**AflII** (8086, 7462, 5659, 5230, 5218 left end, 4285, 2979, 2718, 1620, 1352, 1196 right end)

**BbvCI** (23652 left end, 22153 right end)

### Annotated genome map and phylogenetic analysis

**Fig. 5:**
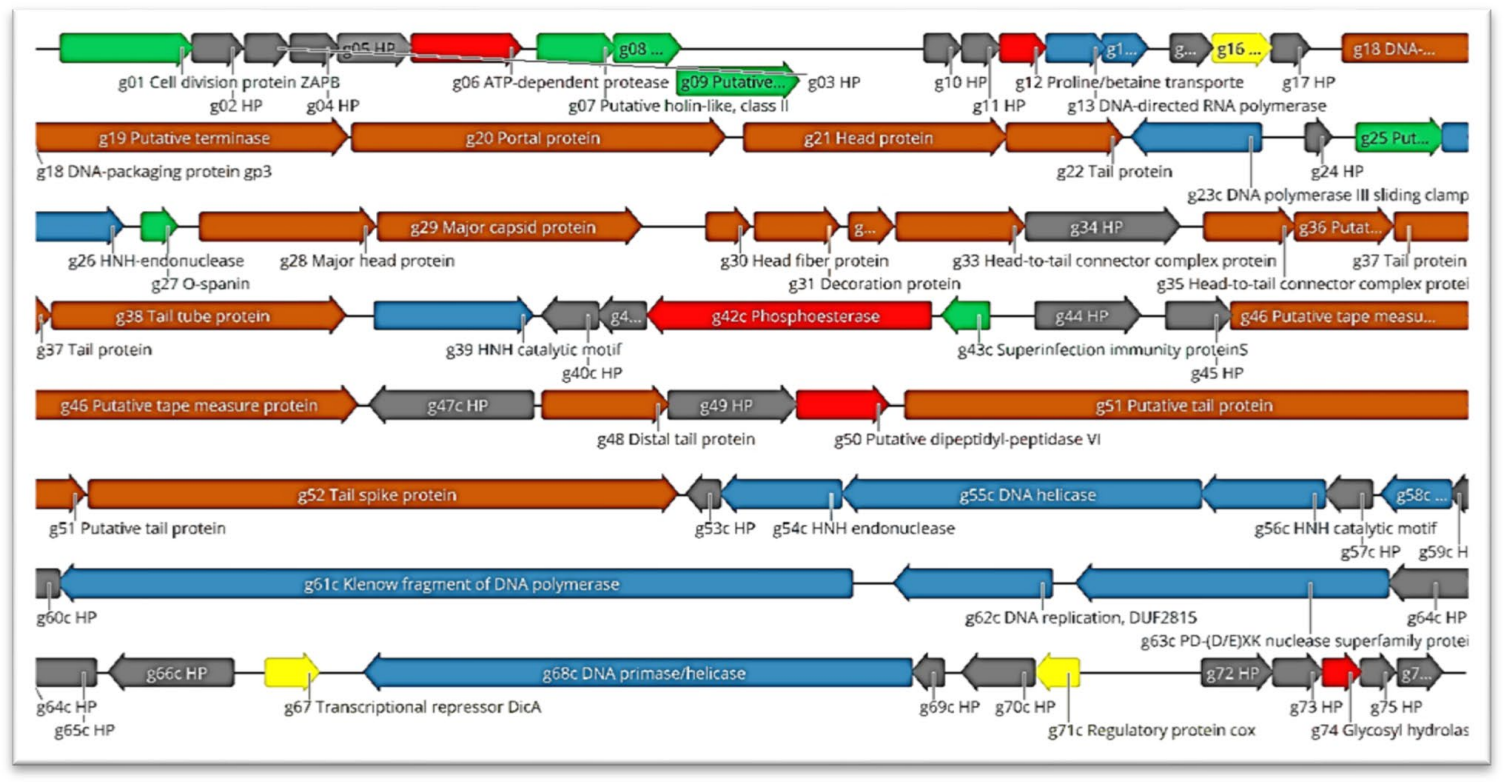
The annotated genome map of fEg-Eco19. The predicted genes are shown as colored arrows labelled with predicted functions (genes encoding structural proteins, brown; DNA/RNA-manipulating proteins, blue; hypothetical proteins, grey; other enzymes, red; lysis functions, green; regulatory proteins, yellow. The map was drawn with Geneious 10.2.6 (www.geneious.com). HP, hypothetical protein; pr, protein

**Fig. 6:**
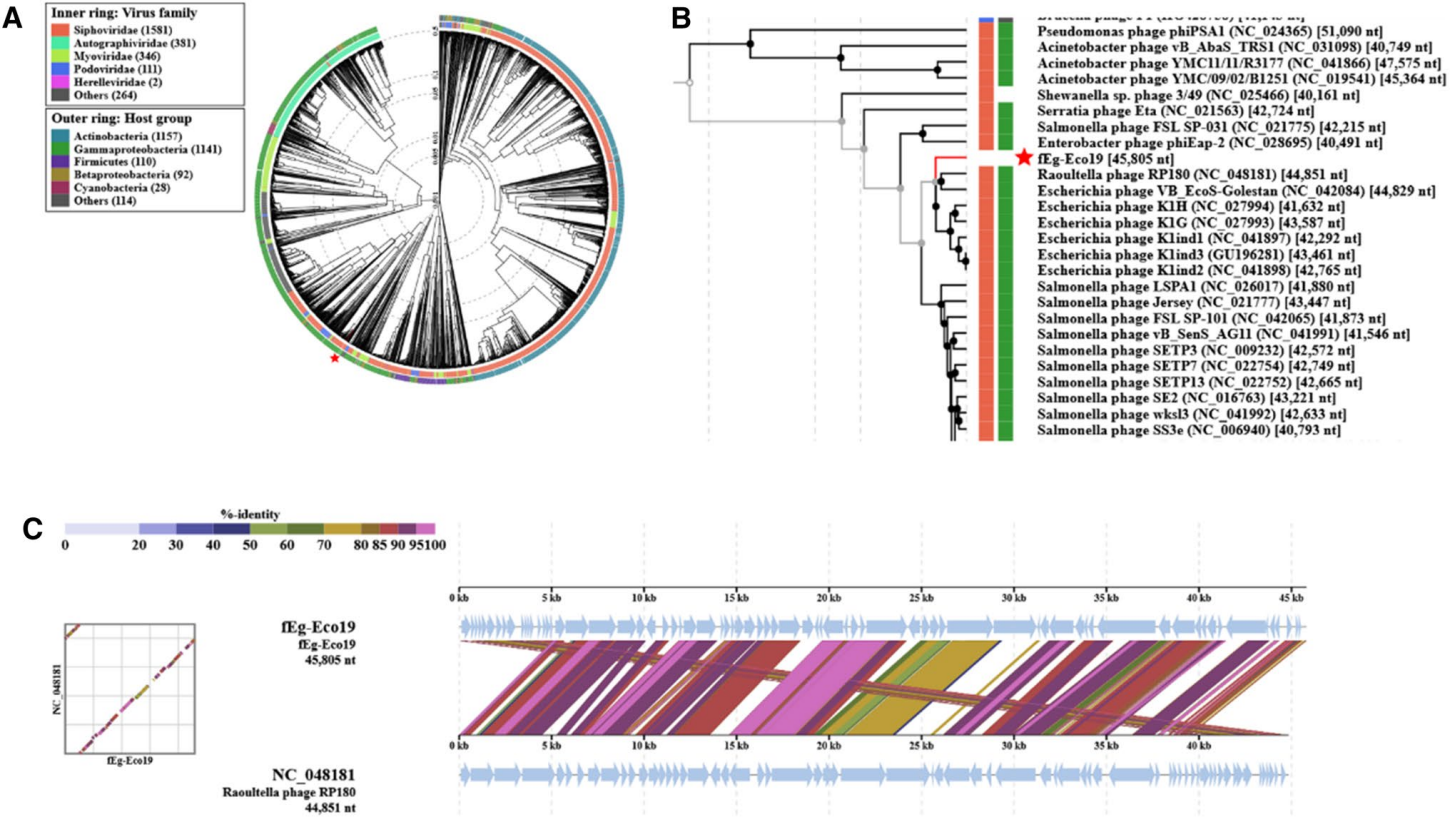
Position of fEg-Eco19 in a phage proteomic tree generated using VIPTree [42] (accessed on Nov. 15, 2021). (A) A circular proteomic tree of prokaryotic dsDNA viruses colored according to virus family and host taxonomic group (B) Part of the rectangular presentation of the proteomic tree showing the phages most closely related to fEg-Eco19. The location of fEg-Eco19 in both is indicated by a red asterisk. (C) Alignment of the fEg-Eco19 genome sequence with that of Raoultella phage RP180

Annotation of the sequences showed that the fEg-Eco19 genome contains 76 predicted genes and has a GC content of 50.2%. The overall organization of the genome of fEg-Eco19 is presented in Figure 5. A BLASTn search of the databases identified a number of *Escherichia* phages that are related to fEg-Eco19, with fFiEco02 (accession no. MT711523) being the closest, with 93.07% identity and 80% query coverage. On the other hand, phylogenetic analysis carried out using VIPtree showed fEg-Eco19 to be most closely related to Raoultella phage RP180 (accession no. NC_048181) and a number of *Escherichia* phages, all with siphovirus morphology (Fig. 6), although the BLASTn search showedfEg-Eco19 to have 90.28% identity to RP180, with 73% query coverage.

### Predicted functions of the phage gene products

The predicted functions of the phage gene products (Gps), based on database searches (Supplementary Table S2), revealed the presence of the following functional groups:

**Regulatory proteins**: A transcriptional regulator (Gp16), a transcriptional repressor (DicA, Gp67), a regulatory protein (cox, Gp71c), and superinfection immunity protein (Gp43c) likely involved in gene regulation [31] were identified.

**DNA packaging and phage structural proteins:** Gp18 is the homolog of the DNA-packaging protein gp3 complex of *Bacillus* phage ϕ29, which is the packaging substrate to which DNA must attach for efficient DNA packaging [32]. DNA is packaged into the cavity of a preformed protein shell, called the prohead, through the connector located at the portal vertex, with the aid of a non-capsid protein called "terminase" or "packaging enzyme" [33, 34]. Packaging of DNA into the phage head is carried out by the putative terminase (Gp19), which recognizes the *cos* site, where it introduces nicks to generate the cohesive ends of the genome and separates the cohesive ends in a reaction requiring ATP hydrolysis [35]. The terminase and the phage portal proteins (Gp20) are believed to be the initiators of head assembly. The phage structural proteins are encoded by genes in the left half of the genome (Fig. 5) and include Gp21, Gp28, and Gp29, which are annotated as head protein, major head protein, and major capsid protein, respectively, and initiate formation of the procapsid [36]. Gp30 is the head fiber protein, Gp31 is the decoration protein, Gp32 is the head-tail joining protein, Gp33 is the head-to-tail connector complex protein, and Gp35 is the head-to-tail connector complex protein. Gp46 is a putative tape measure protein, Gp22 and Gp37 are annotated as tail proteins, Gp36 is a putative tail protein, and Gp38 is a putative tail tube protein. Gp48 is annotated as a distal tail protein, Gp51 as a putative tail protein, and Gp52 as a tail spike protein (Supplementary Table S2).

**Host lysis proteins:** HHpred analysis detected some similarity between Gp1 and the cell division protein ZAPB, which is an abundant cell division factor required for proper Z-ring formation. It is recruited early to the site of division by direct interaction with FtsZ, stimulating Z-ring assembly and thereby promoting cell division earlier in the cell cycle. Its recruitment to the Z-ring requires functional FtsA or ZipA [37]. Gp7 and GP8 are predicted to be holin-like class II and holin-like class I proteins, respectively. Gp9 is predicted to be a putative endolysin functioning as a phage-encoded peptidoglycan hydrolase that breaks down the bacterial peptidoglycan at the end of the reproduction cycle to release the viral progeny [38]. Endolysins have significant advantages over classical antibiotics, including narrow host specificity, high sensitivity, and low probability of development of resistance [39]. Gp25 is predicted to be a spanin, which is a lysis protein that is required for outer membrane disruption. Most phages produce a two-component spanin complex. Gp27 is predicted to be the o-spanin, an outer membrane lipoprotein, and Gp25 is predicted to be the inner membrane protein (i-spanin), which contains a predominantly coiled-coil periplasmic domain. Spanins play an essential role in lysis downstream of the holin-endolysin steps [40].

**DNA/RNA-manipulating proteins and replication gene products:** Gp13 was predicted to be the DNA-directed RNA polymerase (RNAP), which catalyzes the transcription of DNA into RNA using the four ribonucleoside triphosphates as substrates. Classification of phage transcriptional regulatory mechanisms is primarily based on the presence or absence of a phage RNAP. Gp14 was predicted to be the DNA-directed RNA polymerase subunit alpha, which plays an important role in subunit assembly, since its dimerization is the first step in the sequential assembly of subunits to form the holoenzyme [41]. Gp23 was predicted to be the DNA polymerase III sliding clamp, also known as a β-clamp, a protein complex that promotes DNA replication. As a major component of the DNA polymerase III holoenzyme, the clamp protein binds DNA polymerase and prevents this enzyme from dissociating from the template DNA strand. The clamp-polymerase protein–protein interactions are stronger and more specific than the direct interactions between the polymerase and the template DNA strand. The presence of the DNA clamp can increase the rate of DNA synthesis up to 1,000-fold compared with a non-processive polymerase [42]. The HNH endonucleases (Gp26 and Gp54) can nick the double-stranded DNA and may play a variety of roles in replication, recombination, repair pathways, and pathogenicity [31, 43]. In addition, catalytic HNH motifs were identified in Gp39 and Gp56c. Gp58c is annotated to be a restriction endonuclease. Gp61c shows similarity to the T4 DNA polymerase Klenow fragment. Gp62c is annotated to be involved in DNA replication. Gp63c contains a DUF2815 domain and is predicted to be a PD-(D/E)XK nuclease superfamily protein. These proteins are involved in numerous nucleic acid cleavage events that are important for various cellular processes [44]. The DNA helicases Gp55c and Gp68c unwind the DNA to create a template for DNA replication [45].

**Other enzymes**: Gp6 is predicted to be an ATP-dependent protease, a universal barrel‐like, ATP‐fueled machine that prevents the accumulation of aggregated proteins and regulates the proteome according to the demands of the cell. These proteases are distinguished by two separate operating units, the ATPase and peptidase domains. ATP‐dependent unfolding and translocation of a substrate into the proteolytic chamber is followed by ATP‐independent degradation [46]. Gp12 is predicted to be a proline/betaine transporter, a proton symporter that senses osmotic shifts and responds by importing osmolytes such as proline, glycine, betaine, stachydrine, pipecolic acid, ectoine, and taurine. It is both an osmosensor and an osmoregulator that is available to participate early in the bacterial osmoregulatory response [47]. Gp42 is predicted to be a phosphoesterase that, during phage infection, might negatively regulate the growth of the phage, and perhaps of the host as well [48]. Gp50 is annotated to be a putative dipeptidyl peptidase VI. Gp74 is homologous to glycoside hydrolases with lysozyme activity (EC 3.2.1.17). This family includes the lambda phage lysozyme and *E. coli* endolysin [49]. Lysozyme helps mature phage particles to be released from the cell by breaking down the peptidoglycan of the cell wall. It hydrolyses 1,4-beta linkages between *N*-acetyl-D-glucosamine and *N*-acetylmuramic acid in peptidoglycan heteropolymers of prokaryotic cell walls. *E. coli* endolysin also functions in bacterial cell lysis and acts as a transglycosylase.

## Conclusion

In this work, we characterized the *Escherichia coli*-infecting phage fEg-Eco19, which was originally isolated from a sewage sample in Egypt. The phage genome is 45,805 bp in length, containing 76 predicted genes. Based on electron microscopy and phylogenetic analysis, it belongs to the order *Caudovirales* and morphologically resembles siphoviruses. Phage fEg-Eco19 was able to infect only two of the 137 clinical *E. coli* strains tested, and none of the *S. aureus*, *P. aeruginosa*, *A. baumannii*, and *K. pneumoniae* strains tested, indicating that it has a narrow host range. The genome of phage fEg-Eco19 showed a high level of sequence similarity (87.99% identity) to the *Escherichia* phage vB_EcoS_HSE2 (accession no. MG252615.1). Restriction digestion patterns allowed rough mapping of the physical ends of the phage genome, which were identified exactly using the PhageTerm tool. Annotation of the predicted genes revealed gene products of several functional groups, including regulatory proteins, DNA packaging and phage structural proteins, host lysis proteins, and proteins involved in DNA/RNA metabolism and replication.

## Supplementary Information

Below is the link to the electronic supplementary material.

Supplementary file1 (PDF 1032 KB)
