# Supplementary material for: Biological and molecular characterization of fEg-Eco19, a lytic bacteriophage active against an antibiotic-resistant clinical Escherichia coli isolate
Source: Arch Virol. 2022 Apr 10;167(5):1333–41. doi: 10.1007/s00705-022-05426-6 (PMC9038960; doi:10.1007/s00705-022-05426-6)
Supplement: Supplementary file 1 — Supplementary file1 (PDF 1032 KB) [file 705_2022_5426_MOESM1_ESM.pdf]

Supplementary Information

Biological and molecular characterization of fEg-Eco19, a lytic bacteriophage active against antibiotic resistant clinical *Escherichia coli* isolate

Shimaa Badawy [1,2], Zakaria A. M. Baka [2], Mohamed I. Abou-Dobara [2], Ahmed K. A. El-Sayed [2] and Mikael Skurnik [1,3*]

[1] *Department of Bacteriology and Immunology, Medicum, Human Microbiome Research Program, Faculty of Medicine, University of Helsinki, 00014 UH, Helsinki, Finland; shimaa_a_badwy@yahoo.com (S.B.);*

[2] *Department of Botany and Microbiology, Faculty of Science, Damietta University, 34511 New Damietta, Egypt; zakariabaka@du.edu.eg (Z.A.M.B.); aboudobara@du.edu.eg (M.I.A.-D.); akaelsayed@du.edu.eg (A.K.A.E.-S.)*

[3] *Division of Clinical Microbiology, Helsinki University Hospital, HUSLAB, 00290 Helsinki, Finland;*

[*] Correspondence: mikael.skurnik@helsinki.fi; Tel.: +358-50-3360981

Table S1. Bacterial strains used in the work.

Species	Storage Number	Enrichment pool	Resistance Phenotype & Origin[a]	Source[a]
Escherichia coli	5506	Pool 1	Blood	Huslab
	5507	Pool 1	Blood	Huslab
	5509	Pool 1	Blood	Huslab
	5510	Pool 1	Blood	Huslab
	5512	Pool 1	Blood	Huslab
	5517		Blood	Huslab
	5519		Blood	Huslab
	5520	Pool 1	Blood	Huslab
	5521	Pool 1	Blood	Huslab
	5522	Pool 2	Blood	Huslab
	5626		Urine	Huslab
	5627		Urine	Huslab
	5629		Abdominal punction fluid	Huslab
	5632		Urine	Huslab
	5633		Rectum mucus	Huslab
	5634		Rectum mucus	Huslab
	5636		Urine	Huslab
	5639		Urine	Huslab
	5759		ESBL, stool	Huslab
	5760		ESBL, stool	Huslab
	5761		ESBL, blood	Huslab
	5762		ESBL, stool	Huslab
	5763		ESBL, urine	Huslab
	5764		ESBL, urine	Huslab
	5765		ESBL, urine	Huslab
	5766		Abscess in gluteal cleft	Huslab
	5767		Foot surgical wound	Huslab
	5768		ESBL, rectum slime	Huslab
	5769		ESBL, rectum slime	Huslab
	5770		ESBL, Human excretion	Huslab
	6040		ESBL, Human stool	Huslab
	6041		ESBL, Human stool	Huslab
	6042		ESBL, Human stool	Huslab
	6043		ESBL, Human stool	Huslab
	6044		ESBL, Human stool	Huslab
	6045		ESBL, Human stool	Huslab
	6046		ESBL, Human stool	Huslab
	6047		ESBL, Human stool	Huslab
	6048		ESBL, Human stool	Huslab
	6049		ESBL, Human stool	Huslab
	6050		ESBL, Human stool	Huslab
	6051		ESBL, Human stool	Huslab
	6052		ESBL, Human stool	Huslab
	6053		ESBL, Human stool	Huslab
	6054		ESBL, Human stool	Huslab
	6055		ESBL, Human stool	Huslab
	6056		ESBL, Human stool	Huslab
	6057		ESBL, Human stool	Huslab
	6058		ESBL, Human stool	Huslab
	6059		ESBL, Human stool	Huslab
	6060		ESBL, Human stool	Huslab

6061	ESBL, Human stool	Huslab
6062	ESBL, Human stool	Huslab
6063	ESBL, Human stool	Huslab
6064	ESBL, Human stool	Huslab
6065	ESBL, Human stool	Huslab
6066	ESBL, Human stool	Huslab
6067	ESBL, Human stool	Huslab
6068	ESBL, Human stool	Huslab
6070	ESBL, Human stool	Huslab
6071	ESBL, Human stool	Huslab
6072	ESBL, Human stool	Huslab
6073	ESBL, Human stool	Huslab
6074	ESBL, Human stool	Huslab
6075	ESBL, Human stool	Huslab
6076	ESBL, Human stool	Huslab
6077	ESBL, Human stool	Huslab
6078	ESBL, Human stool	Huslab
6079	ESBL, Human stool	Huslab
6080	ESBL, Human stool	Huslab
6081	ESBL, Human stool	Huslab
6082	ESBL, Human stool	Huslab
6083	ESBL, Human stool	Huslab
6084	ESBL, Human stool	Huslab
6085	ESBL, Human stool	Huslab
6086	ESBL, Human stool	Huslab
6087	ESBL, Human stool	Huslab
6088	ESBL, Human stool	Huslab
6089	ESBL, Human stool	Huslab
6090	ESBL, Human stool	Huslab
6091	ESBL, Human stool	Huslab
6092	ESBL, Human stool	Huslab
6093	ESBL, Human stool	Huslab
6094	ESBL, Human stool	Huslab
6095	ESBL, Human stool	Huslab
6096	ESBL, Human stool	Huslab
6097	ESBL, Human stool	Huslab
6098	ESBL, Human stool	Huslab
6099	ESBL, Human stool	Huslab
6100	ESBL, Human stool	Huslab
6101	ESBL, Human stool	Huslab
6102	ESBL, Human stool	Huslab
6103	ESBL, Human stool	Huslab
6104	ESBL, Human stool	Huslab
6105	ESBL, Human stool	Huslab
6106	ESBL, Human stool	Huslab
6107	ESBL, Human stool	Huslab
6108	ESBL, Human stool	Huslab
6109	ESBL, Human stool	Huslab
6110	ESBL, Human stool	Huslab
6111	ESBL, Human stool	Huslab
6112	ESBL, Human stool	Huslab
6113	ESBL, Human stool	Huslab
6114	ESBL, Human stool	Huslab
6115	ESBL, Human stool	Huslab
6116	ESBL, Human stool	Huslab
6117	ESBL, Human stool	Huslab
6118	ESBL, Human stool	Huslab
6119	ESBL, Human stool	Huslab

	6120		ESBL, Human stool	Huslab
	6121		ESBL, Human stool	Huslab
	6122		ESBL, Human stool	Huslab
	6123		ESBL, Human stool	Huslab
	6124		ESBL, Human stool	Huslab
	6125		ESBL, Human stool	Huslab
	6126		ESBL, Human stool	Huslab
	6127		ESBL, Human stool	Huslab
	6128		ESBL, Human stool	Huslab
	6129		ESBL, Human stool	Huslab
	6130		ESBL, Human stool	Huslab
	6131		ESBL, Human stool	Huslab
	6132		ESBL, Human stool	Huslab
	6133		ESBL, Human stool	Huslab
	6134		ESBL, Human stool	Huslab
	6135		ESBL, Human stool	Huslab
	6136		ESBL, Human stool	Huslab
	6137		ESBL, Human stool	Huslab
	6138		ESBL, Human stool	Huslab
	6575	Pool 2	ESBL,103244	DSMZ
	6576	Pool 2	ESBL,103248	DSMZ
	6577	Pool 2	ESBL, 103251	DMSZ
	6578	Pool 2	APEC 2248, Chicken peritoneum	DSMZ
	6581	Pool 2	APEC 9062, Dove	DSMZ
	6741		ESBL, Urine	Huslab
	6742		ESBL, Blood	Huslab
	6882		UPEC, Human acute cystitis	USA
	6883		EPEC, Infant diarrhea	Germany
Pseudomonas aeruginosa	5538		MDR, urine	Huslab
	5539		MDR, urine	Huslab
Acinetobacter baumannii	5542		MDR, Not known	Huslab
	5707		MDR, hip surgical wound	Huslab
Staphylococcus aureus	5696		MRSA, wound pus	Huslab
	5697		MRSA, vagina	Huslab
	5698		MRSA, pharynx	Huslab
	5699		MRSA, pharynx	Huslab
	5700		MRSA, pharynx	Huslab
	5701		MRSA, blood	Huslab
	5702		MRSA, pharynx	Huslab
	5703		MRSA, pharynx	Huslab
	5704		MRSA, pharynx	Huslab
	5705		MRSA, abscess in gluteal cleft	Huslab
Klebsiella pneumoniae	5772		Urine	Huslab

[a] APEC: Avian pathogenic *E. coli*; DSMZ: (Deutsche Sammlung von Mikroorganismen und Zellkulturen GmbH) German Collection of Microorganisms and Cell Culture GmbH; ESBL: Extended Spectrum Beta-Lactamase ; HUSLAB: Hospital District of Helsinki and Uusimaa Laboratories; UPEC: Uropathogenic strains of *E. coli*

Table S2. Annotations of the phage fEg-Eco19 gene products.

fEg-Eco19				Functions predicted by BLASTP (Acc.no) and HHPRED (PDB ID) searches					
Gp	Genomic location	AA	MW	Predicted function	Best hit	BLASTP evalue (Identity)	Query Recovery (%)	HHPRED e-value (probability)	Organism
g01	129:653F	174	20114	cell division protein ZAPB	2JEE_D			1.2(93.51)	*Escherichia coli*
g02	650:853F	67	7410	hypothetical protein BPS11Q3_25	YP_009322832.1	3e-45(100)	100		Salmonella phage BPS11Q3
g03	856:1038F	60	6888	Hypothetical protein EcoSRo145clw_00049	AUX83724.1	8e-39 (96.67)	100		Escherichia phage vB_EcoS-Ro145clw
g04	1038:1232F	64	7632	Hypothetical protein CPT_Snoke_068	QEG07014.1	3e-38(95.31)	100		Escherichia phage Snoke
g05	1229:1522F	97	11407	hypothetical protein	YP_009597323.1	2e-66(96.91)	100		Escherichia phage K1ind1
g06	1519:1956F	145	15401	ATP-dependent protease	YP_009821891.1	3e-95(94.52)	100		Escherichia phage vB_EcoS_XY2
g07	2016:2324F	102	10633	putative holin-like, class II	YP_009168828.1	4e-67(98.04)	100		Escherichia phage K1-dep(4)
g08	2317:2589F	90	10157	putative holin-like, class I	YP_009597374.1	6e-63(100)	100		Escherichia phage K1-ind(2)
g09	2567:3055F	162	17065	putative endolysin	YP_009597375.1	3e-114(97.52)	100		Escherichia phage K1-ind(2)
g10	3548:3697F	49	5352	hypothetical protein L_33	AQN31858.1	9e-29(97.96)	100		Escherichia phage L AB-2017
g11	3694:3849F	51	6029	hypothetical protein GT372_00065	QIG59332.1	2e-31(96.08)	100		Escherichia phage vB_EcoS_XY2
g12	3846:4034F	62	7357	Proline/betaine transporter	1R48_A			0.31(93.12)	N/A
g13	4027:4266F	79	8920	DNA-directedRNA polymerase (E.C.2.7.7.6)	4QIW_W			0.55(86.75)	*Thermococcus kodakarensis*
g14	4253:4435F	60	6720	DNA-directedRNApolymerase subunit alpha	4LLG_N			4.4(86.95)	*Escherichia coli*
g15	4523:4693F	56	6689	hypothetical protein G_30	AQN31787.1	1e-36 (98.21)	100		Escherichia phage G AB-2017
g16	4690:4926F	78	8945	transcriptional regulator	QIG59224.1	5e-51(96.10)	98		Escherichia phage vB_EcoS_XY1
g17	4923:5078F	51	5802	Hypothetical protein GT372_00060	QIG59327.1	3e-32(100)	100		Escherichia phage vB_EcoS_XY2
g18	5206:5751F	181	20073	DNA-packaging protein gp3	3P9A_H			7e-14(99.55)	Enterobacteria phage P22
g19	5748:6998F	416	45928	putative terminase	AUX83735.1	0(99.28)	100		EscherichiaphagevB_EcoSRo145clw
g20	7011:8492F	493	54661	portal protein	QEG06791.1	0(97.56)	99		Escherichia phage Schulenburg
g21	8562:9605F	347	38520	head protein	AUE23503.1	0(96.54)	100		Escherichia phage vB_EcoS_HSE2
g22	9605:10066F	153	16301	tail protein	QIG59322.1	3e-104 (96.73)	100		Escherichia phage vB_EcoS_XY2
g23c	10094:10615R	173	19627	DNA polymerase III sliding clamp	QEG06795.1	4e-124(96.53)	100		Escherichia phage Schulenburg
g24	10786:10896F	36	4162	hypotheticalprotein CPT_Shashou_007	QEA09405.1	6e-21(100)	100		Escherichia phage Shashou
g25	10991:11350F	119	13150	putative spanin	YP_009620087.1	4e-68(82.91)	98		EscherichiaphageVB_EcoSGolestan

g26	11334:11840F	168	19109	HNH-endonuclease	1U3E_M			6.2e-13(99.34)	Bacillus phage SPO1
g27	11909:12055F	48	5505	o-spanin	QEA09407.1	2e-29 (100)	100		Escherichia phage Shashou
g28	12138:12839F	233	25423	Major head protein	6B0X_e			0.036(97.03)	Staphylococcus phage 80alpha
g29	12842:13891F	349	37836	major capsid protein	6R3A_B			1.5e-49(100)	Bacillus phage SPP1
g30	14145:14321F	58	5671	Head fiber protein	3QC7_A			6.3e-8(98.55)	Bacillus phage phi29
g31	14333:14674F	113	11562	decoration protein	QEG06885.1	2e-65(90.27)	100		Escherichia phage Sciku
g32	14711:14890F	59	6681	head-tail joining protein	QIG59212.1	7e-37(98.31)	100		Escherichia phage vB_EcoS_XY1
g33	14894:15406F	170	17682	head-to-tail connector complex protein	QIG59211.1	1e-116 (95.88)	100		Escherichia phage vB_EcoS_XY1
g34	15409:16023F	204	20750	Hypothetical protein Schulenberg_017	QEG06805.1	3e-138(96.08)	100		Escherichia phage Schulenburg
g35	16118:16477F	119	12917	head-to-tail connector complex protein	QIG59311.1	3e-80 (95.80)	100		Escherichia phage vB_EcoS_XY2
g36	16474:16869F	131	14541	putative tail protein	AQN31838.1	2e-92(96.95)	100		Escherichia phage L AB-2017
g37	16869:17282F	137	14999	tail protein QIG59309.1	QIG59309.1	2e-97 (97.81)	100		Escherichia phage vB_EcoS_XY2
g38	17285:18451F	388	40843	Tail tube protein	5NGJ_A			4e-16(99.77)	Escherichia phage T5
g39	18559:19191F	210	24027	HNH catalytic motif	1U3E_M			0.00025(97.41)	Bacillus phage SPO1
g40	19220:19450R	76	8681	Hypothetical protein Schulenberg_023	QEG06811.1	3e-51 (98.68)	100		Escherichia phage Schulenburg
g41c	19440:19640R	66	7554	Hypothetical protein Schulenberg_024	QEG06812.1	1e-43 (100)	100		Escherichia phage Schulenburg
g42c	19637:20767R	376	42345	phosphoesterase	QEG06813.1	0(98.67)	100		Escherichia phage Schulenburg
g43c	20806:20997R	63	7357	Superinfection immunity protein	QEG06897.1	8e-38(96.83)	100		Escherichia phage Sciku
g44	21176:21595F	139	15592	hypotheticalprotein vBEcoSRo145clw_00015	AUX83757.1|	4e-96(97.12)	100		Escherichia phage vB_EcoS-Ro145clw
g45	21694:21960F	88	10117	hypotheticalprotein vBEcoSRo145clw_00016	AUX83758.1	6e-62(100)	100		Escherichia phage vB_EcoS-Ro145clw
g46	21953:24226F	757	81030	putative tape measure protein	AQN31947.1	0(97.23)	100		Escherichia phage P AB-2017
g47c	24270:24920R	216	25134	Hypothetical protein GT371_00005	QIG59196.1	3e-154(97.22)	100		Escherichia phage vB_EcoS_XY1
g48	24956:25456F	166	18285	Distal tail protein	6F2M_D			0.05(95.96)	Escherichia phage T5
g49	25453:25968F	171	19076	hypothetical protein L_2	AQN31827.1	4e-121(100)	100		Escherichia phage L AB-2017
g50	25965:26330F	121	13996	putative dipeptidyl-peptidase VI	3NPF_A			1.2e-9(99.13)	*Bacteroides ovatus*
g51	26393:28876F	827	90966	putative tail protein	YP_009168859.1	0(98.07)	100		Escherichia phage K1-dep(1)
g52	28889:31225F	778	81907	Tail spike protein	6EU4_C			2.2e-23(99.94)	Acinetobacter phage vB_AbaP_AS12
g53c	31256:31393R	45	5184	hypotheticalprotein	AUX83698.1	2e-23(97.78)	100		Escherichia phage vB_EcoS-Ro145clw

				vBEcoSRo145clw_00022					
g54c	31390:31875R	161	18487	HNH endonuclease	YP_009191564.1	5e-112(95.65)	100		Salmonella phage f18SE
g55c	31872:33296R	474	53492	DNA helicase	YP_008239745.1	0(96.41)	100		Salmonella phage Jersey
g56c	33289:33786R	165	18626	HNH catalytic motif	1U3E_M			5.4e-14(99.54)	Bacillus phage SPO1
g57c	33783:33974R	63	7071	hypothetical protein	YP_009608589.1	5e-40(100)	100		Salmonella phage vB_SenS_AG11
g58c	34005:34292R	95	10903	restriction endonuclease	YP_009821870.1	2e-64(96.84)	100		Raoultella phage RP180
g59c	34279:34377R	32	3958	Hypothetical protein vse101_38	QFR58890.1	5e-17(93.75)	100		Salmonella virus VSe101
g60c	34374:34505R	43	4701	hypothetical protein GT371_00068	QIG59259.1	1e-21(93.02)	100		Escherichia phage vB_EcoS_XY1
g61c	34495:37647R	1050	118460	KLENOW fragment of DNA Polymerase	2KFZ_A	2.8e-52(100)	100		*Escherichia coli*
g62c	37805:38437R	210	23594	DNA replication, DUF2815	5ODJ_A	1.5e-31(100)	100		Enterobacter phage Enc34}
g63c	38527:39768R	413	45888	PD-(D/E)XK nuclease superfamily protein	QIN98015.1	0(95.64)	100		Salmonella phage pink
g64c	39765:40112R	115	13342	hypothetical protein	QIG57327.1	4e-80(96.52)	100		Salmonella phage vB_SpuP_Spp11
g65c	40109:40381R	90	10697	hypothetical protein LSPA1_43	YP_009113190.1				Salmonella phage LSPA1
g66c	40425:40925R	166	18793	hypothetical protein Schulenberg_049	QEG06837.1	3e-108(96.39)			Escherichia phage Schulenburg
g67	41050:41265F	71	7697	transcriptional repressor DicA	AQN31878.1	3e-44(100)	100		Escherichia phage L AB-2017
g68c	41440:43611R	723	82980	DNA primase/helicase	6N7I_B	3.4e-16(99.73)			Enterobacteria phage T7
g69	43608:43736R	42	5040	hypothetical protein G_51	AQN31808.1	7e-24(100)	100		Escherichia phage G AB-2017
g70c	43805:44095R	96	11317	hypothetical protein GT372_00012					Escherichia phage vB_EcoS_XY2
g71c	44092:44274R	60	7153	Regulatory protein cox; helix-turn-helix,	4LHF_A			2.9e-7(98.72)	Enterobacteria phage P2
g72	44758:45039F	93	10204	hypothetical protein RP180_46	YP_009821882.1	2e-47(91.14)	84		Raoultella phage RP180
g73	45039:45236F	65	7448	hypothetical protein RP180_47	YP_009821883.1	3e-34(86.89)	93		Raoultella phage RP180
g74	45233:45388F	51	6011	glycosyl hydrolase	AQN31870.1	2e-30(98.04)	100		Escherichia phage L AB-2017
g75	45385:45528F	47	5589	hypothetical protein GT371_00050	QIG59241.1	5e-26(93.62)	100		Escherichia phage vB_EcoS_XY1
g76	45532:45717F	61	6976	hypothetical protein E1_58	YP_009620138.1	7E-36(91.53)	96		Escherichia phage VB_EcoS-Golestan

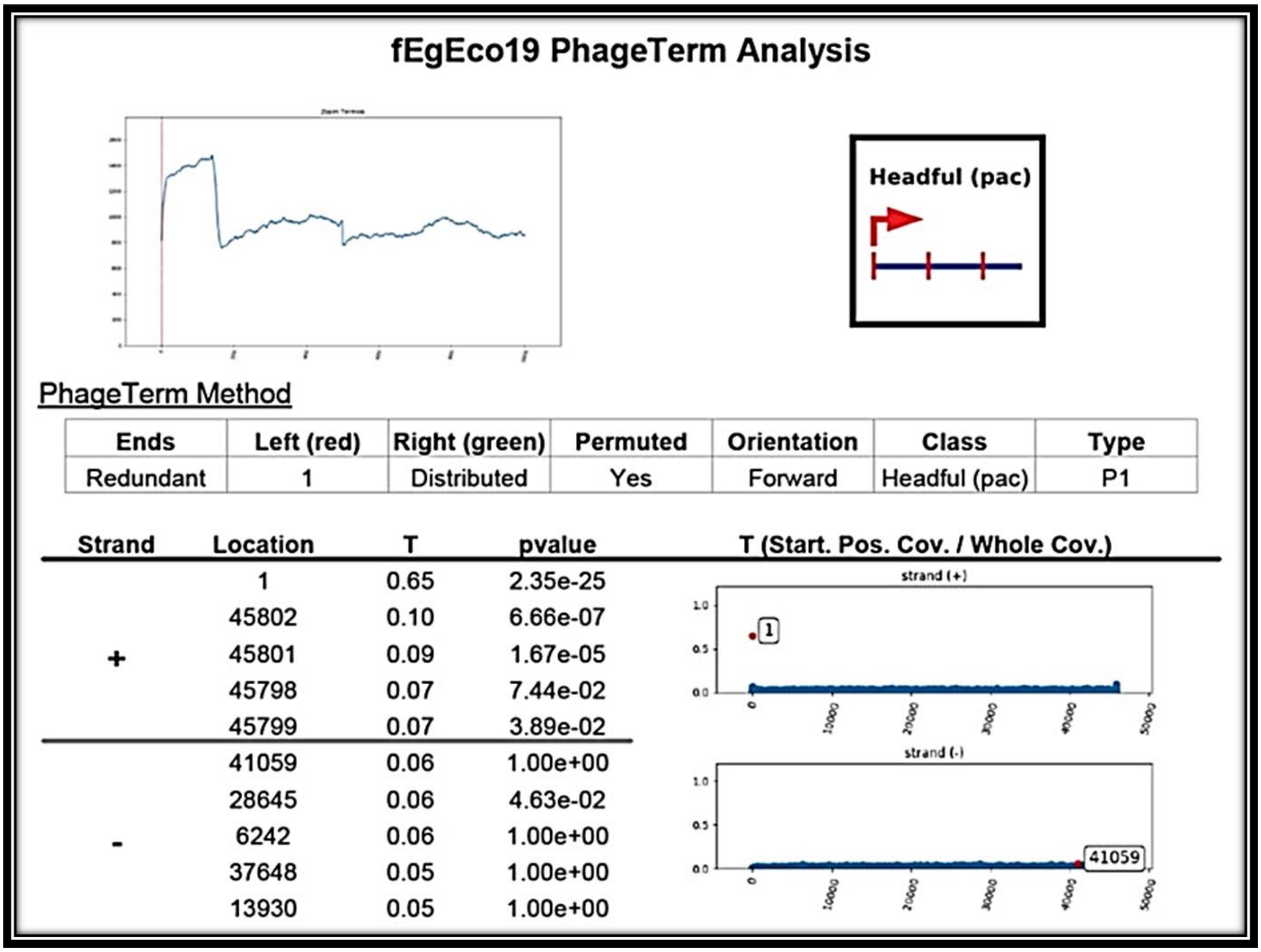

Ends	Left (red)	Right (green)	Permuted	Orientation	Class	Type
Redundant	1	Distributed	Yes	Forward	Headful (pac)	P1

Strand	Location	T	pvalue
+	1	0.65	2.35e-25
	45802	0.10	6.66e-07
	45801	0.09	1.67e-05
	45798	0.07	7.44e-02
	45799	0.07	3.89e-02
-	41059	0.06	1.00e+00
	28645	0.06	4.63e-02
	6242	0.06	1.00e+00
	37648	0.05	1.00e+00
	13930	0.05	1.00e+00

Figure S1. PhageTerm analysis of the fEg-Eco19 sequence read data demonstrating the presence of a headful packaging mechanism and redundant ends.
